# Supplementary material for: Examination of the efficacy of small genetic panels in genomic conservation of companion animal populations
Source: Evol Appl. 2020 Jun 19;13(10):2555–65. doi: 10.1111/eva.13038 (PMC7691451; doi:10.1111/eva.13038)
Supplement: Supplementary file 1 — Supplementary Material [file EVA-13-2555-s001.zip › eva13038-sup-0002-AppendixS2.docx]

**Appendix B - Supplementary Figures**


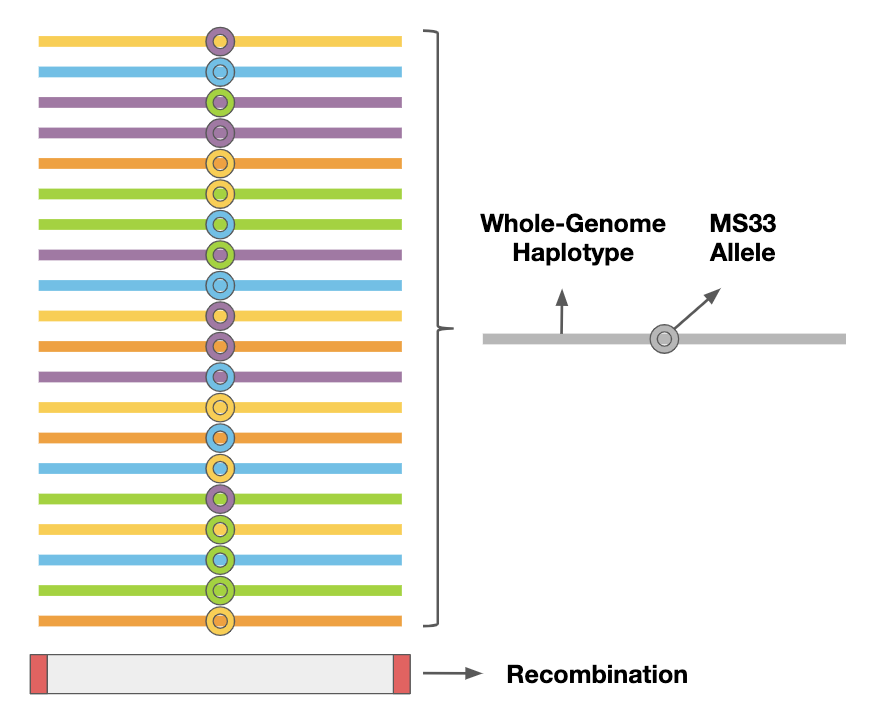


**Figure B1. Illustration of initialization state at a haplotype locus carrying a microsatellite marker**

Bars represent whole genome haplotype markers. Circles represent microsatellite markers. Colors represent alleles. Each whole genome haplotype marker is bounded by points of recombination.


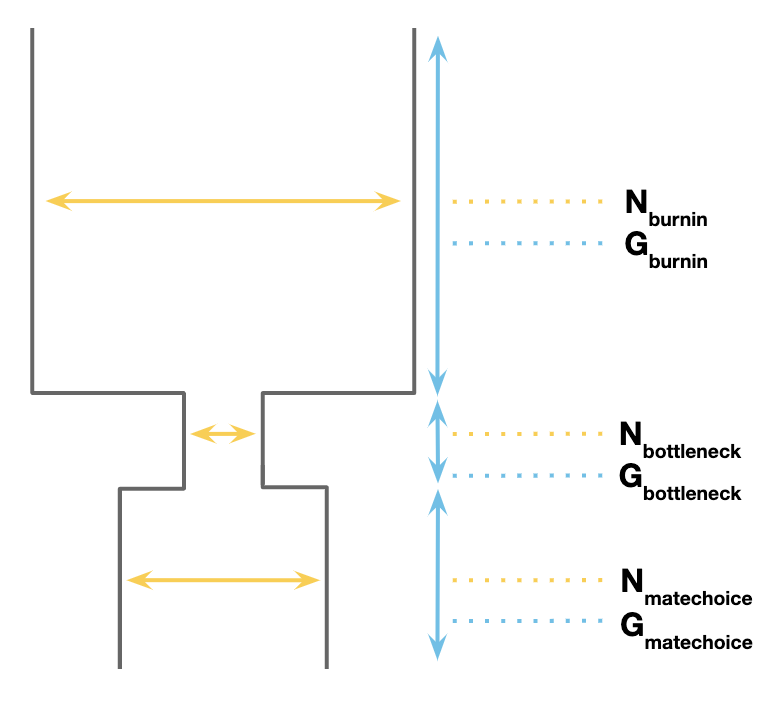


**Figure B2. Illustration of demographic model**

N – Population sizes, G – Number of generations.


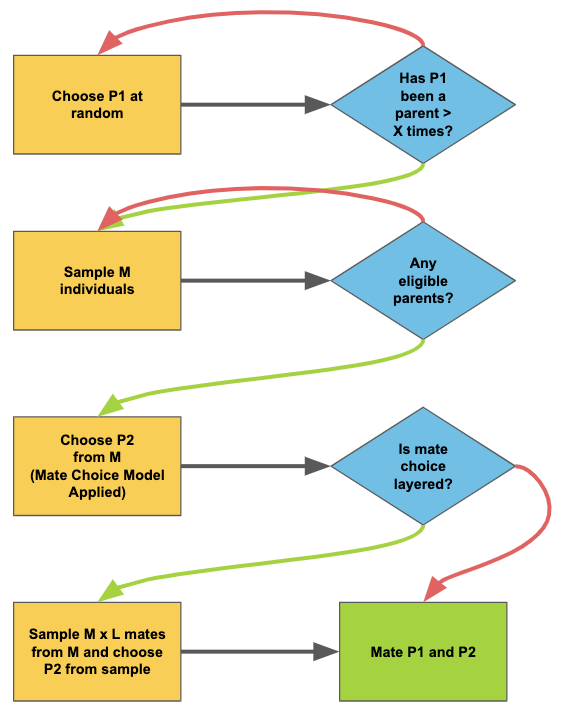


**Figure B3. Flow chart of mate choice life cycle**

Yellow boxes represent sampling events, blue diamonds represent decision points, green box represents final state (both parents chosen).

P1 — Parent 1

P2 — Parent 2

M — Random sample of individuals with size = MATING_POOL_SIZE

X — Count of number of times within generation that P2 has been used as a parent (restricted to <= MAXIMUM_NUMBER_OF_MATINGS)

L — Proportion of mating pool to sample from in layered mate choice schemes = PROPORTION_OF_MATES_FOR_LAYERED_MATE_CHOICE


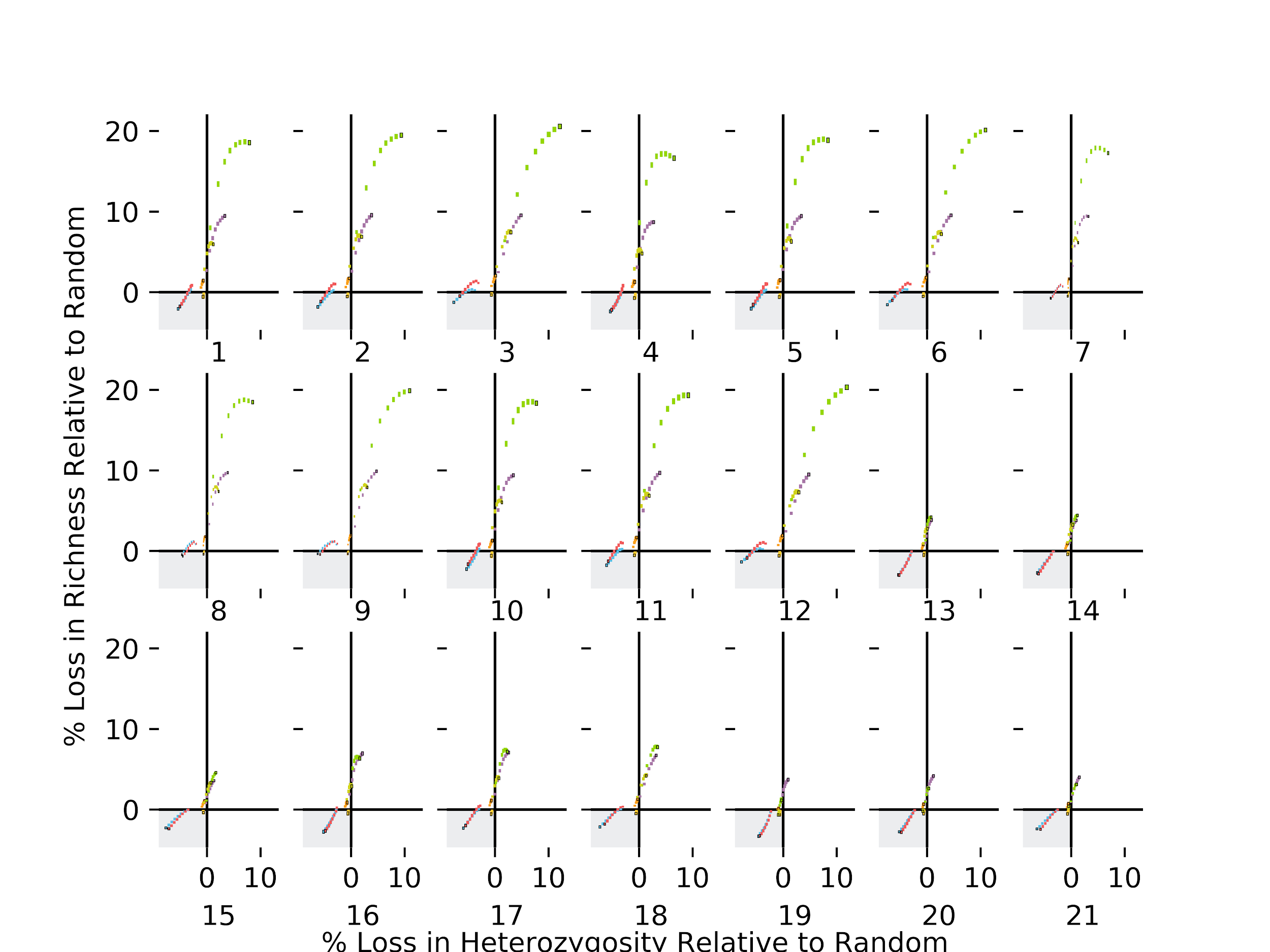


**Figure B4. Percent loss in heterozygosity and richness across all simulations**

Parameter set key is as in Figure 1.
